# Understanding key features of bacterial restriction-modification systems through quantitative modeling

**DOI:** 10.1186/s12918-016-0377-x

**Published:** 2017-02-24

**Authors:** Andjela Rodic, Bojana Blagojevic, Evgeny Zdobnov, Magdalena Djordjevic, Marko Djordjevic

**Affiliations:** 1 Institute of Physiology and Biochemistry, Faculty of Biology, University of Belgrade, Studentski trg 16, 11000 Belgrade, Serbia; 2 Multidisciplinary PhD program in Biophysics, University of Belgrade, Belgrade, Serbia; 3 Institute of Physics Belgrade, University of Belgrade, Belgrade, Serbia; 4 Department of Genetic Medicine and Development, University of Geneva and Swiss Institute of Bioinformatics, Geneva, Switzerland

**Keywords:** Restriction-modification, Transcription regulation, Bacterial immune systems, Biophysical modeling, Gene expression dynamics

## Abstract

**Background:**

Restriction-modification (R-M) systems are rudimentary bacterial immune systems. The main components include restriction enzyme (R), which cuts specific unmethylated DNA sequences, and the methyltransferase (M), which protects the same DNA sequences. The expression of R-M system components is considered to be tightly regulated, to ensure successful establishment in a naïve bacterial host. R-M systems are organized in different architectures (convergent or divergent) and are characterized by different features, i.e. binding cooperativities, dissociation constants of dimerization, translation rates, which ensure this tight regulation. It has been proposed that R-M systems should exhibit certain dynamical properties during the system establishment, such as: *i*) a delayed expression of R with respect to M, *ii*) fast transition of R from “OFF” to “ON” state, *iii*) increased stability of the toxic molecule (R) steady-state levels. It is however unclear how different R-M system features and architectures ensure these dynamical properties, particularly since it is hard to address this question experimentally.

**Results:**

To understand design of different R-M systems, we computationally analyze two R-M systems, representative of the subset controlled by small regulators called ‘C proteins’, and differing in having convergent or divergent promoter architecture. We show that, in the convergent system, abolishing any of the characteristic system features adversely affects the dynamical properties outlined above. Moreover, an extreme binding cooperativity, accompanied by a very high dissociation constant of dimerization, observed in the convergent system, but absent from other R-M systems, can be explained in terms of the same properties. Furthermore, we develop the first theoretical model for dynamics of a divergent R-M system, which does not share any of the convergent system features, but has overlapping promoters. We show that *i*) the system dynamics exhibits the same three dynamical properties, *ii*) introducing any of the convergent system features to the divergent system actually diminishes these properties.

**Conclusions:**

Our results suggest that different R-M architectures and features may be understood in terms of constraints imposed by few simple dynamical properties of the system, providing a unifying framework for understanding these seemingly diverse systems. We also provided predictions for the perturbed R-M systems dynamics, which may in future be tested through increasingly available experimental techniques, such as re-engineering R-M systems and single-cell experiments.

**Electronic supplementary material:**

The online version of this article (doi:10.1186/s12918-016-0377-x) contains supplementary material, which is available to authorized users.

## Background

Restriction-modification systems are rudimentary bacterial immune systems, whose main components are the restriction enzyme (R), and the methyltransferase (M). We here consider Type II restriction-modification (R-M) systems [1], where R cuts the same DNA sequences that are protected by M. Consequently, R and M act, respectively, as a toxic molecule and its antidote, and analogies of R-M and toxin-antitoxin systems are often made [2]. R-M present rudimentary “bacterial immune systems”, as they protect the host bacterial cell against infection by foreign DNA, such as viruses (bacteriophages) [3–6]. The protection mechanism is straightforward, as the foreign DNA entering bacterial cell is unmethylated, and is consequently cut (destroyed) by R. On the other hand, the host DNA is methylated due to presence of M, and is therefore not cut by R, which prevents autoimmunity. In fact, many bacteriophages are under pressure from R-M systems with whom they have common hosts [7, 8], and have developed different mechanisms to avoid restriction [9–11]. Consequently, expression of the toxic molecule and its antidote provides an effective protection of the bacterial cell against foreign DNA infection [12].

R-M systems are often mobile [2, 12, 13], spreading from one bacterial host to the other, so that a bacterial host, which initially did not contain the R-M system (a naïve host), can acquire it through horizontal transfer. Expression of R and M was directly observed in single cells only very recently, for the Esp1396I system [14], and it is still unclear how different R-M system features affect this expression. It is however assumed that R-M expression has to be tightly regulated during its establishment in a naïve host [15]. For example, as the naïve host genome is initially unmethylated, R must be, and where tested actually is, expressed after a delay with respect to M, so that the host’s genomic DNA can be protected before the appearance of R [14, 16, 17]. To ensure such tight regulation, a significant subset of R-M systems contains a third gene, which expresses the control protein (C) [5, 6, 18–23]. C is a transcription factor, which regulates expression of genes in R-M system, including its own expression. In fact, C is typically co-transcribed with R from a common promoter (CR promoter), while M is transcribed from a separate promoter (M promoter) [5, 6, 24].

**Fig. 1:**
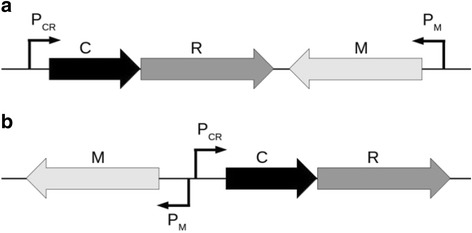
Typical gene arrangement and promoter orientation in convergent and divergent R-M systems. **a** Convergent systems, a representative of which is AhdI, where other studied systems encoding C protein include Esp1396I, Kpn2I, Csp231I, PvuII [14, 23, 47–49]. Note that C and R genes are transcribed together from P_CR_ promoter. Transcription of M is exhibited from the separate P_M_ promoter. **b** Divergent systems, a representative of which is EcoRV, where BamHI is another studied divergent system that encods C protein [20]. C and R genes are also co-transcribed, but now share a common promoter region with M gene. In EcoRV the two divergent promoters (P_CR_ and P_M_) have overlapping RNA polymerase binding sites

With respect to the organization of the transcription units, two different architectures are exhibited, which correspond to the convergent (Fig. 1a), and the divergent (Fig. 1b) orientation of CR and M promoters [5, 6, 14, 20, 21, 23, 25, 26]. Despite R-M systems being known for few decades now, with numerous biotechnological uses of restriction enzymes, control of expression of these systems has been insufficiently studied. Two relatively well studied examples are AhdI (a representative of the convergent architecture) [6], and EcoRV (a divergent architecture representative) [5]. For both systems, the core promoters (binding sites of RNA polymerase), and the binding sites of C protein, are experimentally mapped. In addition, for AhdI system, the transcription activity of CR promoter was measured as a function of C protein amount. We previously showed that a thermodynamic model of CR promoter regulation provides a good agreement with this measurement [6]. We also recently showed [14] that a similar thermodynamic model, coupled with a dynamical model of transcript and protein synthesis, can reasonably explain the dynamics of the enzyme synthesis measured by single-cell experiments in another convergent R-M system (Esp1396I). This strongly suggests that quantitative modeling presented here can realistically explain R-M system transcription control. Additionally, thermodynamical modeling of transcription regulation was successfully applied to a number of different biological problems [27–30], while dynamical modeling was applied to explain both more and less complex gene circuits including control of other convergent R-M systems [31–33].

As we detail below on the example of AhdI (convergent system), and EcoRV (divergent system), it is experimentally firmly established that R-M systems exhibit both different architectures, and different features that characterize their gene expression regulation [1, 15]. On the other hand, the regulation should yield the same three dynamical properties, so that the host genome is protected, while the system is efficiently established. In particular, as discussed above, there would have to be a significant expression of M before R is expressed, to ensure that the host genome is protected. Furthermore, once the host genome is protected, the system should likely turn to “ON” state as rapidly as possible, so that the host genome becomes “immune” to the virus infections – this would then require that after an initial delay, R is rapidly generated. Finally, we also previously proposed that, once the toxic molecule (R) reaches a steady-state, its fluctuations should be low – otherwise a high fluctuation in the toxic molecule (R) may not be matched by the antidote (M), which could destroy the host genome [34].

It is however unclear how the diverse system features and architectures, relate with the constraints on the dynamical response of the system stated above. Experimentally, one could, in principle, address this issue by mutating the relevant features (or introducing them in the system where they do not exist), and then measuring how the resulting system dynamics is perturbed. This would however be very hard, as the system would have to be extensively experimentally mutated and/or redesigned, and the resulting protein dynamics measured in-vivo during the system establishment. In that respect, note that the in-vivo dynamics of R and M expression were directly observed for only two Type II systems – in PvuII via nearly simultaneous introduction into a culture using bacteriophage M13 [17], and in Esp1396I, via transformation followed by single cell analysis [14]. Even in these cases, the measurements are done only on the wild-type (wt) system, i.e. perturbations were not introduced in the system.

Therefore, the main purpose of this paper is to investigate the relationship between different system features/architectures, and the dynamical properties which the system is expected to exhibit during its establishment. In particular, it is our hypothesis that the diverse features exhibited in R-M systems may largely be explained in terms of the three dynamical properties discussed above. To start testing this hypothesis, we will here biophysically model the control of AhdI and EcoRV, and assess the resulting dynamics when the characteristic system features are either perturbed (in AhdI case) or (artificially) introduced (in EcoRV case) in the system. This is analogous to a classical approach in molecular biology, where the system is analyzed by mutating its main features, or introducing new features in the system where they do not exist, and consequently observing what effect these perturbations have on the presumed system function. The difference is that we here analyze the system *computationally* instead of *experimentally*, where we build on the fact that we previously showed that the modeling approach that we employ here can reasonably explain the available equilibrium measurements [6], and the available single cell experiments [14]. Therefore, the ability of the modeling to explain the measured wild-type data in R-M systems provides a reasonable confidence that our predictions for the perturbed system will also be realistic. Moreover, with the advancement of sophisticated experimental approaches, such as single cell experiments, or possibility to reengineer the system, there comes a prospect of directly experimentally testing these predictions in the future.

Specifically, we will here start by reviewing the relevant experimental information for AhdI and EcoRV systems (the structure of their promoter regions and their regulatory features), which will provide a bases for our theoretical modeling. We will then quantify the general principles discussed above, i.e. introduce what we here call the dynamical property observables, which will allow us quantifying the delay between R and M, how fast the system makes the transition from OFF to ON state, and the stability of R steady-state levels. We will then investigate if abolishing the characteristic features of AhdI also diminishes these observables, i.e. negatively affects the dynamical properties discussed above. Furthermore, we will also study if these dynamical properties also apply to the system (EcoRV) where AhdI features are absent, but a new feature is present (the overlapping promoters). We will then ask what happens if the AhdI features are (computationally) introduced in wild-type EcoRV system, where they originally do not exist. That is, we will investigate if introducing these features leads to (at least) some of the three dynamic property observables being diminished – therefore explaining why they are absent from EcoRV. Overall, we will here systematically investigate how perturbing (or introducing new) features in two characteristic R-M systems affects the resulting system dynamics.

## Methods

In the first subsection, we provide in detail the experimentally available information on AhdI (the convergent system) and EcoRV (the divergent system), on which we base our quantitative modeling. The main properties of the model, including the observables through which we assess the system dynamical properties, are provided in the second subsection. We note that the model itself is provided in details in Additional files 1 and 2, where all the parameters (including their experimental/theoretical support) are listed.

### Experimentally determined configurations of AhdI and EcoRV

For AhdI, the positions of different promoter elements (C protein and RNAP binding sites) were experimentally mapped for both CR and M promoters [6] (see Fig. 1a). In addition, the binding affinities and the transcription activities for both the wild type and mutant systems (where C protein binding sites were mutated) were measured [6]. These measured values, together with the standard literature values for the kinetic parameters (the translation and the degradation rates), were used to parameterize the model, as provided in detail in Additional file 1.

**Fig. 2:**
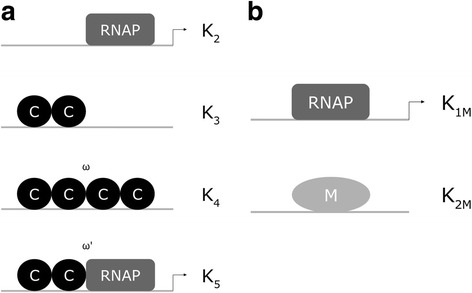
AhdI R-M system promoter regions. The arrangement of the promoter elements for AhdI CR and M promoters is based on the experimental information provided in [6]. The regions which are schematically shown correspond to (**a**) P_CR_ promoter. Circles indicate C monomers, the rectangles indicates RNAP, while the arrows indicate transcriptionally active configurations. K_2_ – K_5_ denote the dissociation constants (see Additional file 1) corresponding to different promoter configurations (K_1_ denotes the dissociation constant of dimerization), where ω and ω’ denote, respectively, the binding cooperativity between the two C dimers bound to DNA, and between C dimer bound to the distal binding site and RNAP. C binds to the promoter as either dimer (K_3_) or tetramer (K_4_). The bound dimer recruits RNAP to the promoter (K_5_). On the other hand, the tetramer configuration corresponds to the repression, as it prevents RNAP binding to the promoter. **b** Transcription is repressed by DNA methylation due to M binding [6], i.e. M methylates specific sites in M promoter that overlap RNAP binding site – for simplicity this is in the figure represented as M being bound to the promoter DNA

As indicated in Fig. 2a, C binds to CR promoter, regulating both its own transcription and the transcription of R [6, 19]. C binds to promoter DNA as a dimer, where binding to the distal binding site (configuration K_3_), when C is present at relatively low concentration, leads to transcription activation, as C dimer bound to this position recruits RNAP binding to the promoter (configuration K_5_). On the other hand, when C is present at high concentration, C dimer bound to the distal binding site recruits another C dimer to the proximal binding site (the tetramer configuration, K_4_), thus repressing the transcription, as RNAP cannot bind to the promoter. Note that the configuration in which C dimer binds only to the proximal binding site (equivalent to K_3_) is not shown, as the binding affinity to the proximal binding site is much lower compared to the distal binding site, making this configuration much less probable. As for M gene, its transcription is controlled by a negative feedback loop, i.e. M methylates specific sites in its own core promoter thereby repressing the transcription (Fig. 2b).

There are three features which characterize control of AhdI expression [6]. First, there is a very high cooperativity in binding of the C protein dimers to the distal and the proximal positions in CR promoter, so that C dimer bound only to the distal site (K_3_ configuration) exists only very transiently in the wild-type (wt) AhdI system. That is, in the absence of RNAP, a C dimer bound to the distal position immediately recruits another C dimer to the proximal binding site. Second, the C dissociation constant of dimerization for AhdI is very high, so that almost all C protein in the solution is in the form of monomers. Finally, C protein is translated from a leaderless transcript (i.e. a transcript which does not contain a ribosome binding site), which was in *E. coli* shown to be associated with lower translation initiation rate [35, 36].

For EcoRV, CR and M promoters are divergently oriented, as schematically shown in Fig. 1b. Consequently, the promoter elements are located in the intergenic region that separates CR and M genes, and these elements are also experimentally mapped [5]. Some of the binding affinities were also measured [5], while the others were eliminated by rescaling the equations (see Additional file 2) – note that we can rescale the equations, as we are interested only in the relative protein amounts. The kinetic parameters (the translation and the degradation rates), correspond to the standard literature values, and are taken to be the same as for AhdI (with the exception of C translation rate, see below).

**Fig. 3:**
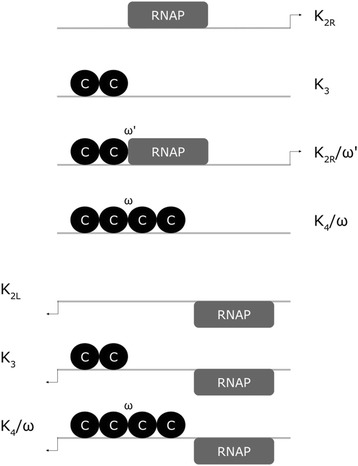
Transcription regulation of EcoRV R-M system. The promoter configuration in the figure is based on the experimentally mapped promoter elements from [5]. Note that the promoters for CR (P_CR_) and M (P_M_) genes are divergent, as schematically shown in Fig. 1b. C and R genes are co-transcribed from the rightward promoter (P_CR_, see Fig. 1b), with RNAP bound to the promoter as indicated in the first and the third configuration (from top to bottom). M gene is transcribed from the divergent P_M_ promoter (see Fig. 1b), with RNAP bound to the promoter as indicated in the last three configurations. P_M_ and P_CR_ core promoters partially overlap each other, so that RNAP cannot simultaneously bind to P_M_ and P_CR_. The explanations for the first four configurations are equivalent as in Fig. 2a. Note that *ω*′ denotes the binding cooperativity between the dimer bound at the distal position and RNAP. For the last three configurations, note that binding of C does not directly influence binding of RNAP to P_M_ [5]

In contrast to AhdI, the main feature of EcoRV is the partially overlapping CR and M core promoters, as schematically shown in Fig. 3. Consequently, RNAP cannot simultaneously bind to and initiate transcription from both P_M_ and P_CR_. Moreover, the characteristic features of AhdI are not found in EcoRV [5]. In particular, while the transcription control of the CR promoter by C protein is similar as in AhdI, the main difference is that the large cooperativity between the C dimers at the distal and the proximal binding site is now absent, in fact it was found in EcoRV that the two dimers bind to DNA with no cooperativity [5]. Furthermore, the transcription from P_M_ is not directly influenced by C protein binding, i.e. C binding does not directly affect RNAP binding to P_M_. However, the influence of C on P_M_ transcription is indirect, as the regulation by C of RNAP binding to P_CR_, also affects when RNAP can bind to P_M_. Consequently, while in AhdI transcription of CR and M was independent from each other, in EcoRV we have a more complex system where their transcription is strongly coupled. Similar regulation through overlapping CR and M core promoters is also found in CfrBI R-M system [26, 37]. Finally, C transcript is not leaderless in EcoRV, so the feature which was associated with lower translation initiation rate in *E. coli*, and which is present in AhdI, is now absent from EcoRV.

### Modeling AhdI and EcoRV dynamics

We model R and M synthesis upon introducing AhdI and EcoRV in naïve bacterial hosts. The models are based on the experimental knowledge of AhdI and EcoRV transcription regulation, which is summarized in Figs. 2 and 3, respectively. The models are provided in detail in Additional files 1 and 2, and are briefly based on:

(i) A thermodynamic model, which takes into account the activation and the repression of CR promoter by C, and the repression of M gene by its own product (which was experimentally shown in [6]). The model assumes that the promoter transcription activity is proportional to the equilibrium binding probability of RNAP to promoter, which is a general assumption initially proposed by the classical Shea-Ackers approach [38].
(ii) Equations that predict how the transcription activity of CR and M promoters depends on C-protein concentration, which further allows modeling the dynamics of transcript and protein expression. That is, the modeled transcription activities provide the main input for a kinetic model, which calculates R, C and M transcript and protein synthesis. Also, note that R-M systems are characterized by very high expression of R and M proteins [14] so that on the order of thousands of molecules are present in the cell. Consequently, the system is expected to be well in the limit where deterministic modeling can be used to realistically describe the system.

We previously showed that such modeling can well explain the wild-type measurements for AhdI [6] - in particular the measured dependence of the transcription activity on C protein concentration – as well as the most recent measurements in single-cell experiments allowing directly observing the dynamics of R and M synthesis [14]. Our aim here is to computationally analyze how systematically abolishing individual system features affects the system’s dynamics, focusing on the following properties:

i. the time delay between R and M accumulation,
ii. the transition speed of the system from “OFF” to “ON”,
iii. the stability of R steady-state levels.

**Fig. 4:**
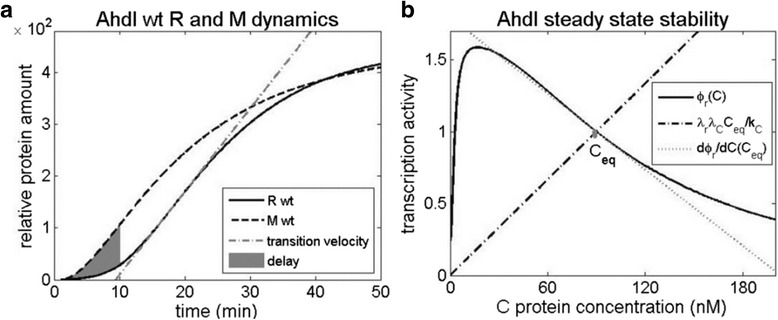
**a** Dynamics of R and M expression. R and M expression upon the system entry in a naïve bacterial host (0 min corresponds to the system entry). The shaded area corresponds to the difference of the surface areas below M (dashed curve) and R (solid curve) expression curves for the first 10 min post-system entry; the area presents a measure of the delay between M and R expression. The dash-dot line corresponds to the maximal slope of the sigmoidal R expression curve, measuring the transition velocity from OFF to ON state. **b** Steady-state and its stability. The steady-state (indicated by C_eq_) is obtained as an intersection of the transcription activity (the solid black line), and the dash-dot line whose slope is determined by the transcript decay and the protein translation rate (Eq. (1.33)). The stability of the steady-state is related with the difference of the dash-dot line slope, and the slope of the transcription activity (the dotted line in the figure) at the point of their intersection C_eq_ (Eq. (1.34))

For this, we will introduce observables (which we call the dynamical property observables) that can quantify these properties. To reasonably define them, it is useful to visualize the predicted system dynamics, and the stability of R steady-state levels in wild-type AhdI system, which is shown in Fig. 4 and calculated from Eqs. (1.12), (1.22) and (1.24)–(1.27) (see Additional file 1).

#### The first dynamical property observable (delay)

From Fig. 4a, we see that the system features lead to a significant delay in the expression of R compared to M, in accordance with the first dynamical property. To quantify how the delay changes upon perturbing these features, we introduce the first dynamical property observable, which corresponds to the ratios of the shaded areas in the perturbed system and in wt AhdI, at an initial interval post-system entry.

#### The second dynamical property observable (OFF to ON transition speed)

Furthermore, in Fig. 4a, we see that R expression curve has a sigmoidal shape. Consequently, the maximal slope of this curve (indicated in the figure) provides a reasonable measure of transition velocity from “OFF” (low R value) to “ON” (high R value) state. Therefore, as the second dynamical property observable, we introduce the maximal slope of this curve. The changes of this slope will allow assessing how the transition velocity – which determines the time window between the host genome being methylated, and the cell being protected against viruses – will be affected when the system features are perturbed.

#### The third dynamical property observable (R steady-state level stability)

Finally, the third dynamical property relates with fluctuations of the toxic R molecule, which we propose should be small in the steady-state [34]. The fluctuations are directly related with the stability of the steady-state, so that smaller fluctuations imply larger steady-state stability, which we introduce as the third dynamical property observable.

Different (in-silico) perturbations of the wild-type system – i.e. gradually abolishing the existing or introducing new features – will be introduced in either the thermodynamic model, or in the kinetic equations (see Additional files 1 and 2).

## Results and discussion

We will start by gradually abolishing the three characteristic AhdI features introduced above, and assess how this will affect the dynamical property observables. We will next model the dynamics of EcoRV establishment in a naïve bacterial host, to see if the proposed dynamical properties also apply to a system with different architecture and transcription regulation features. This will provide, to our knowledge, the first quantitative model of a divergent R-M system control, and an opportunity to assess dynamics of R and M expression, which was up to now not experimentally observed for the divergent systems. Finally, we will in-silico introduce to EcoRV the regulation features that exist in AhdI, but are not found in EcoRV, to investigate how this effects the dynamical property observables, and why these features are not present in EcoRV.

### Perturbing AhdI system features

The three characteristic AhdI features are the high C subunit dissociation constant of dimerization, the large cooperativity between C dimers bound at the distal and the proximal position, and the low C transcript translation initiation rate. It was previously discussed that these features serve to limit the amount of the synthesized toxic molecule (R) [6]. However, it is not clear that this amount per-se should be limited, as a too small steady-state amount of R may compromise the immune response – i.e. it can lead to the virus genome being protected by M before it can be destroyed by R [39]. As we discussed above, it would be very hard to experimentally investigate the effect of these AhdI features on the system dynamics, this can be readily predicted from the model that we formulated above.

### Decreasing the dissociation constant of dimerization

**Fig. 5:**
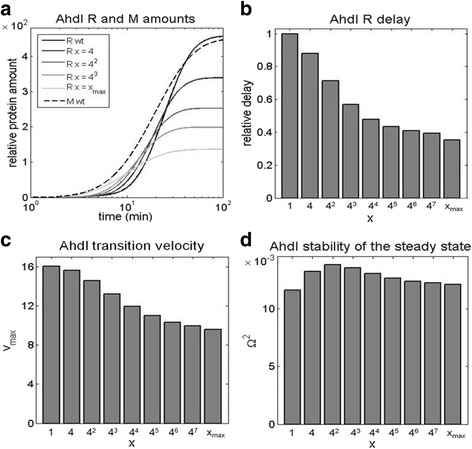
Decreasing AhdI dissociation constant of dimerization. *K*_1_ is decreased from the high value corresponding to mostly monomers in the solution, to the low value corresponding to mostly dimers in the solution, and the effect is assessed on **a** The dynamics of the protein synthesis. The black line corresponds to all monomers in the solution (wt), while the light gray line corresponds to all dimers in the solution. The curves in-between (in different shades of gray) correspond to the gradually decreasing values of *K*_1_. The relative protein amounts for a wt system (on the vertical axis) are derived from in-vitro transcription activity measurements in [6]. x indicated in the legend corresponds to the relative decrease of *K*_1_ (e.g. x = 4 is a four-fold decrease). **b** The first dynamical property observable, corresponding to the relative delay of R with respect to M expression. The delay is normalized with respect to the wild type (corresponding to one). **c** The second dynamical property observable, corresponding to the transition velocity from “OFF” to “ON” state, represented by the maximal slope of the R expression curve. **d** The third dynamical property observable, corresponding to the stability of R steady-state levels (see Methods)

The dissociation constant of dimerization *K*_1_ is very high for AhdI, leading to almost all C subunits being present as monomers in solution [6, 40] – e.g. for another convergent R-M system (Esp1396I), the measured dissociation constant of dimerization was found to be significantly (four times) lower [41]. We start by gradually decreasing this high dissociation constant of dimerization, in the range that corresponds to the wild-type (all monomers in the solution) to the opposite limit of lower *K*_1_, in which only dimers are present in the solution. In Fig. 5a, we see that this perturbation has a significant effect on R synthesis dynamics – note that the M dynamics curve, which is also indicated in the figure for reference, is not affected by perturbing the three characteristic AhdI features. One can observe the three main effects from Fig. 5a: The decrease of the delay between R and M expression, the slower transition from OFF to ON state, and the decrease in the steady-state level of R. The first two effects are further quantified in Fig. 5b and c, as discussed below.

In Fig. 5b, we see that decreasing *K*_1_ leads to a significant, more than twofold, decrease in the relative delay between R and M expression. This perturbation can then significantly impact the ability of the system to protect the host genome from being cut during R-M establishment, with the necessary lag also depending on the specific activity of the M protein and the propensity for R to nick hemimethylated sites. Furthermore, in Fig. 5c we see that decreasing *K*_1_ also leads to a significantly slower transition from OFF to ON state, so that the maximal slope is decreased for almost two-fold. Therefore, decreasing the wt dissociation constant of dimerization also significantly impacts the time window in which the host will be protected from foreign DNA infection. However, perturbing K1 has no significant effect on the steady-state stability of R levels (Fig. 5d). Overall, decreasing the high dissociation constant of dimerization characteristic for wt AhdI, has a significant adverse effect on two of the three proposed design principles.

### Increasing C protein translation rate

**Fig. 6:**
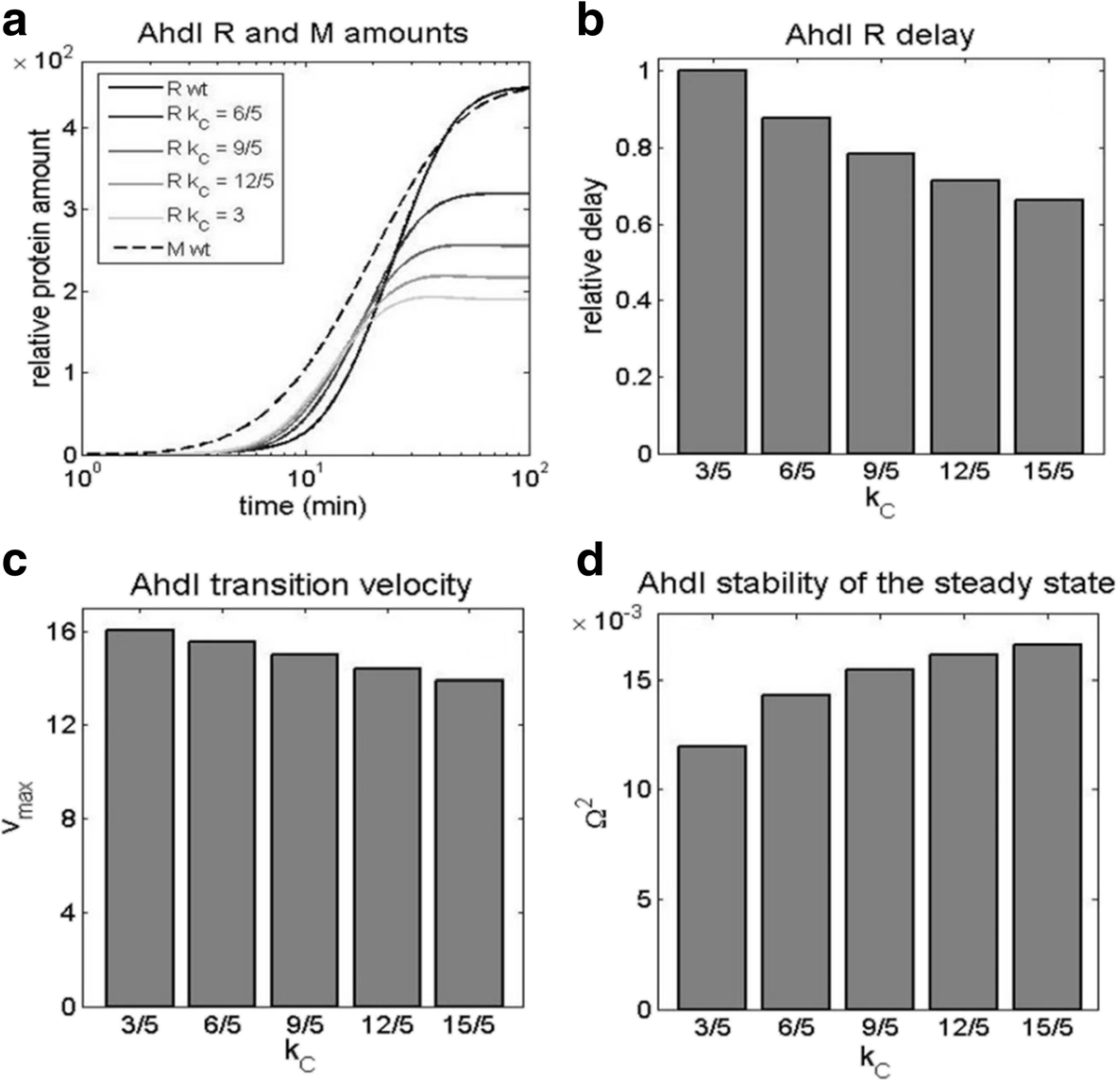
Increasing C transcript translation rate: *k*_C_ is increased from the lower value (3/5 1/min) as taken in [6] to the value which equals those for R and M transcripts (3 1/min). The effect of this decrease is assessed for: **a** The dynamics of the protein synthesis, with the black curve corresponding to the lowest (wt) *k*_C_, and the light gray curve corresponding to the highest *k*_C_ (which equals those of R and M transcripts). The curves in different shades of gray correspond to the gradually increasing *k*_C_ values. **b** The relative delay (normalized with respect to wt) of R with respect to M expression. **c** The maximal slope of the R expression curve, reflecting the transition velocity from “OFF” to “ON” state. **d** The stability of R steady-state levels, is shown on the vertical axis

In AhdI C transcript is leaderless [6], which was in *E. coli* [35, 36] shown to be associated with a significantly smaller translation initiation rate – consequently in [6] a five times smaller C transcript translation rate *k*_C_, compared to R and M was assumed. We now test the effect of perturbing this system feature, i.e. increasing *k*_C_ towards those of R and M transcripts, which is shown in Fig. 6. We see that the main effect of this perturbation is on decreasing the steady-state level of R and the delay between R and M expression (for ~ 40%), as shown in Fig. 6a-b. Intuitively, this can be understood that by a more efficient C transcript translation, C accumulates faster, facilitating the formation of the activating and the repressing complexes on the CR promoter, so that R is expressed with a smaller delay, and reaches the lower steady-state level. On the other hand, the effect on the other two design-observables, i.e. on the transition velocity and the stability of R steady-state levels, is rather small (Fig. 6c-d). Consequently, increasing the low C transcript translation rate adversely affects one of the dynamical property observables, i.e. the delayed expression of R with respect to M, which is considered crucial for the protection of the host genome.

### Decreasing cooperativity in the dimer binding

**Fig. 7:**
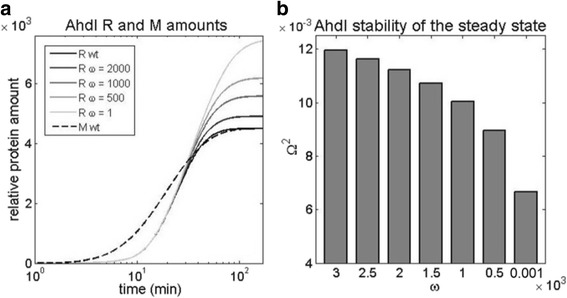
Decreasing cooperativity in C dimer binding to CR promoter. The cooperativity in binding ω is gradually abolished from the very high value corresponding to wt AhdI [6] to *ω* corresponding to the absence of the binding cooperativity. We predict the effect of this decrease on: **a** The dynamics of R protein synthesis, where the black line corresponds to the high *ω*, the light gray to no cooperativity, and the values of cooperativity in-between are shown in different shades of gray. **b** The stability of R steady-state levels, corresponding to different *ω* values shown in **a**

A rather drastic feature of AhdI is a very large cooperativity *ω* in binding of the two dimers to the distal and the proximal position in the promoter [6], which is either not present (EcoRV) [5], or significantly smaller (Esp1396I) [41], in other R-M systems. We therefore investigate how gradually abolishing this high cooperativity affects the system dynamics and the design observables. In Fig. 7a, we see that abolishing *ω* affects only the late dynamics of R, so that the first two dynamical properties are not affected (and not shown in Fig. 7). On the other hand, we see that the steady-state amount of R significantly increases as the cooperativity *ω* decreases. This can be intuitively understood by the fact that perturbing the cooperativity affects only the efficiency of forming the repressor tetramer complex. As the probability of forming this complex is proportional to C^4^ (see Additional file 1), it becomes significant only in the later period, when a large enough amount of C is synthesized. Furthermore, in accordance with the perturbation affecting the late dynamics, from Fig. 7b, we see that decreasing the cooperativity significantly impacts the stability of R steady-state levels, leading to its 50% decrease.

Importantly, the first two AhdI features (the large dissociation constant of dimerization, and the small C translation initiation rate) have an opposite effect on the steady-state amount of R, as compared to the large cooperativity in C dimer binding. That is, while we showed that the first two features significantly increase the steady-state R amount, the third feature (the large cooperativity) significantly decreases it. On the other hand, all three features generally have the same effect on the three dynamical properties that we consider, i.e. abolishing these features either decreases the values of the dynamical property observables (making the corresponding dynamical property less optimal), or do not significantly affect them. This can then explain the extremely large binding cooperativity that was experimentally observed, as on the one side it allows controlling the steady-state amount of the toxic protein due to the opposite effect from the other two features, while at the same time working together with the first two features to ensure more optimal dynamical properties. In particular, note that both the large dissociation constant of dimerization and the large binding cooperativity significantly increase the stability of R steady-state levels, while having a significant - but opposite – effects on the steady-state R amounts.

### EcoRV wild-type dynamics

EcoRV is an example of R-M system with a divergent organization of CR and M transcription units. Overlapping CR and M promoters is the most distinctive feature of this system (presenting its main difference with respect to AhdI), which is, together with C protein binding, responsible for control of EcoRV transcription. That is, high occupancy of M promoter by RNAP, prevents RNAP binding to CR promoter, leading to lower CR transcription activity, and vice versa. In modeling the gene expression regulation, we consider that CR promoter transcription is controlled by C, while C binding has little to none direct effect on M promoter transcription activity, as shown in [5]. In distinction to AhdI [6], which shows an extremely high cooperativity in C dimer binding, no coperativity was found in EcoRV [5]. We also assume that C dissociation constant of dimerization is significantly lower than the relevant range of C concentration, so that the majority of C molecules in solution exist as dimers. Note that in another R-M system (Esp1396I), which has a much lower cooperativity in C dimer binding compared to AhdI, a significantly lower dissociation constant of dimerization is also observed [41]. Finally, in distinction to AhdI, C transcript in EcoRV is not leaderless, so for EcoRV we assume that C has the same translation initiation rate as R and M.

Consequently, EcoRV does not have the three features that control transcription in AhdI, but has instead another characteristic feature, i.e. the overlapping CR and M promoters. We therefore ask if EcoRV, with different architecture and the regulation features, can also meet the three dynamical properties that we consider. To that end, we modeled the synthesis of R and M during the system establishment in wild-type EcoRV, under the assumptions stated above, and following the scheme of the transcription configurations shown in Fig. 3. The model is provided in detail in Additional file 2, and is based on the same thermodynamics assumptions as the one for AhdI dynamics. To our knowledge, this presents the first model of expression dynamics for a divergent R-M system, which has a more complex regulation due to overlapping nature of their promoters. This model moreover presents the first opportunity to assess the dynamics of R and M synthesis for a divergent R-M system, as, to our knowledge, either their regulation or their expression dynamics was not previously measured.

**Fig. 8:**
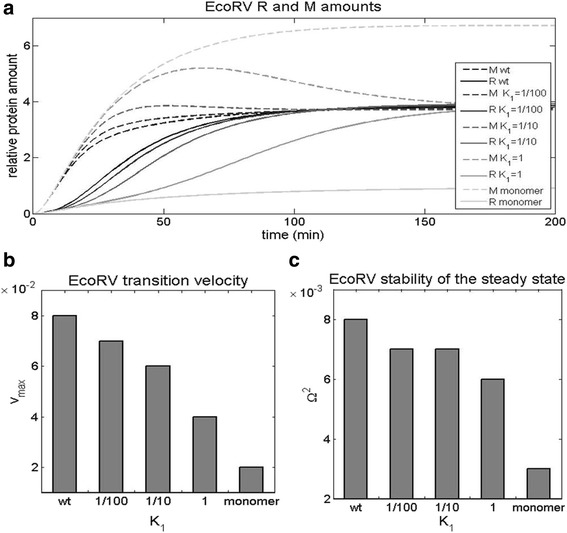
Increasing the dissociation constant of dimerization of wt EcoRV system. The rescaled dissociation constant of dimerization ${\overline{K}}_1$ is increased from the lower value with dimers in the solution corresponding to wt system, to the high value, where mostly monomers are in the solution. The effect of the increasing dissociation constant of dimerization is assessed on: **a** The dynamics of R and M synthesis. The solid and the dashed line correspond to R and M dynamics, respectively. Different shades of gray correspond to the increasing value of the dissociation constant of dimerization, with the black line and the light gray line corresponding to the wild type and the monomer case, respectively. **b** The transition velocity v_max_ from “OFF” to “ON” state. **c** The stability of R steady-state levels

The predictions for R and M accumulation in wild-type EcoRV are shown by the full black curve (for R) and by the black dashed curve (for M), in Fig. 8 below. From the figure we see that, regardless of lacking the characteristic AhdI regulatory features, the synthesis of R and M is well in accordance with the three dynamical properties. Namely, by comparing Fig. 4 (the dynamics of AhdI) with the EcoRV dynamics, we see that: *i*) the time delay for EcoRV is even larger compared to AhdI, *ii*) there is a clear switch-like behavior of R expression in EcoRV, i.e. the speed of transition from “OFF” to “ON” state is comparable to the one in AhdI, iii) the system reaches the steady-state level (Ω^2^ > 0), where the reached stabilities of R steady-state levels are comparable (compare Fig. 5d with Fig. 8c). Therefore, we see that the design principles which we showed are inherent to AhdI R-M system, are retained in EcoRV R-M system, despite the apparent distinction in gene expression regulation.

### Introducing AhdI control features to EcoRV

Next, there is a question of why the characteristic AhdI features are absent from EcoRV. That is, could we get even more optimal design-observables if AhdI control features are introduced in wild-type EcoRV? Therefore, we next use our model, to individually introduce each of the three control features of AhdI, on the top of the existing wt EcoRV regulation (i.e. the overlapping promoters). Specifically, in the wild-type EcoRV, we will perturb: *i*) the dissociation constant of dimerization towards the high values characteristic for AhdI, *ii*) cooperativity in C dimer binding to the promoter, also towards the high values observed in AhdI, *iii*) C protein translation rate *k*_C_, towards the low values characteristic for leaderless AhdI C transcripts.

### Introducing the high dissociation constant of dimerization to EcoRV

We first perturb the wt EcoRV system by increasing the rescaled equilibrium dissociation constant of dimerization ${\overline{K}}_1$ (see Fig.8 and Additional file 2), which corresponds to a gradual transition from the solution containing mostly C dimers to the solution containing mostly C monomers. Note that the dynamics of both R and M expression is now affected by the perturbation, in distinction to AhdI where only R expression is changed. This is because CR and M promoters overlap in EcoRV, so that changing transcription from one promoter, necessarily impacts transcription from the other.

We observe that this perturbation does not significantly affect the early accumulation of R and M (during the first ~10 min), but that the dynamics at later times is significantly affected (see Fig. 8a). In particular, we see that increasing the dissociation constant of dimerization leads to a significantly slower switch from “OFF” to “ON” state, so that the transition velocity decreases as much as four times (Fig. 8b). Furthermore, in Fig. 8c, we see that increasing ${\overline{K}}_1$ also significantly decreases the stability of R steady-state levels Ω^2^, which drops almost three times. Consequently, introducing the high dissociation constant of dimerization to EcoRV, which is characteristic for AhdI, has a significant adverse effect on two of the three dynamical properties.

### Introducing the high C dimer binding cooperativity

**Fig. 9:**
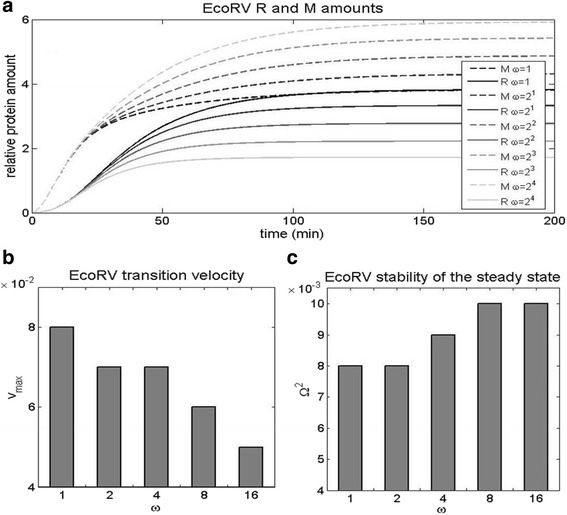
Increasing C dimer binding cooperativity in wt EcoRV. The binding cooperativity ω is increased from the absence of cooperativity (ω = 1, corresponding to wt EcoRV), to the higher values corresponding to cooperative C dimer binding. For each curve, ω is increased in steps by a factor of 2, and the effect is assessed on: **a** The dynamics of R and M synthesis. The solid and the dashed curves correspond, respectively, to the dynamics of R and M synthesis. The black curve corresponds to wt (no cooperativity), with the curves fading, as the cooperativity increases (with the light gray corresponding to maximal ω). **b** The transition velocity v_max_. **c** The stability of R steady-state levels

We next modify wt EcoRV by increasing the cooperativity ω of C dimer binding to the proximal and the distal binding site, while keeping the other wt EcoRV features unchanged. Note that the experimental measurements in wt EcoRV show an absence of C dimer binding cooperativity (ω = 1) [5], as opposed to the extremely large binding cooperativity that is observed in AhdI [6]. In Fig. 9, we see that increasing ω has the following effects: *i*) the time delay remains nearly the same (Fig. 9a), *ii*) the transition velocity decreases (Fig. 9b), where we see that increasing ω for a relatively moderate factor (2^4^), leads to a significant (somewhat less than twofold) decrease of v_max_, *iii*) stability of R steady-state levels slightly increases. Consequently, we see that perturbing wt EcoRV cooperativity towards the higher values characteristic for AhdI, has a significant adverse effect on one of the dynamical properties (the transition velocity), while not significantly affecting the other two.

### Decreasing C translation rate in EcoRV

**Fig. 10:**
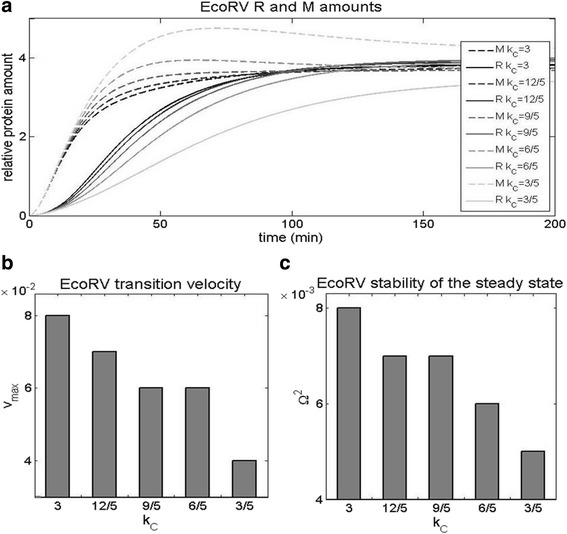
Decreasing C transcript translation rate of wt EcoRV system. The translation rate of C transcript is decreased towards the low value characteristic for wt AhdI, and the effect is assessed on **a** The dynamics of R and M synthesis. The solid curves correspond to R, while the dashed curves correspond to M. The curves fade as *k*_C_ decreases, so that the black curve, and the light gray curve, correspond, respectively, to the maximal (wt) and the minimal *k*_C_. **b** The transition velocity from “OFF” to “ON” state. **c** The stability of R steady-state levels

Finally, we perturb wt EcoRV by decreasing C transcript translation rate k_C_, towards the value characteristic for AhdI. Note that C transcript is leaderless in AhdI [6], which is not the case for EcoRV [5], so that we assume the same translation rate for all three transcripts (C, R and M) in EcoRV, while *k*_C_ is taken as five times lower in AhdI according to [6]. In Fig. 10a we observe that decreasing *k*_C_ does not impact the initial R and M accumulation (during the first ~10 min). On the other hand, at later times the perturbation significantly decreases both the transition velocity that decreases two times (see Fig. 10b), and the stability of R steady-state levels that decreases somewhat less than twofold (see Fig. 10c). Consequently, we see that again two of the three dynamical properties are significantly adversely affected by introducing a control feature from AhdI.

Overall, introducing AhdI characteristic features to EcoRV has a significant adverse effect on at least one of the dynamical properties, which may explain why those features are not found in EcoRV. Additionally, perturbing EcoRV wt parameters towards the AhdI values (Figs. 8a, 9a and 10a) changes M to R ratio in the same direction for each introduced feature (consistently increasing the ratio). This is in distinction to AhdI, where the high cooperativity of C dimer binding has an opposite effect on this ratio, compared to the other two features. Consequently, we argue that another reason for why the characteristic AhdI features are not observed in EcoRV, is because they do not allow balancing the amounts of R and M in the host cell.

## Conclusion

R-M systems are characterized by different architectures and control features. We here test a hypothesis that these diverse features can be explained by constraints imposed by few dynamical properties. We started from a relatively well studied AhdI system, and computationally abolished three of its characteristic control features, showing that this has a clear adverse effect on the three dynamical properties. We then modeled a system with different architecture (EcoRV), and showed that its expression dynamics also satisfies the same properties. The EcoRV model has significance in its own right, as the expression dynamics of the divergent R-M systems was, to our knowledge, not studied before, either theoretically or experimentally. Finally, we computationally introduced to EcoRV the control features that exist in AhdI, and showed that this diminishes at least some of the proposed dynamical properties, consistent with the fact that these features do not appear in wt EcoRV. Moreover, increasing the binding cooperativity has the same effect on M to R ratio in EcoRV as increasing the dissociation constant of dimerization, or lowering the translation rate, which prevents balancing M to R ratio upon introducing these perturbations – this then provides another argument for why AhdI control features are absent from wt EcoRV.

Furthermore, dynamical properties proposed here can provide an explanation for a surprisingly large value of the cooperativity in C protein binding, accompanied by the large dissociation constant of dimerization that was observed in wt AhdI. We here showed that these two features have an opposite effect on the steady-state levels of the toxic molecule (R), allowing balancing the steady-state R amount, while at the same time leading to more optimal dynamical properties. In support of this proposal, a similar convergent system with lower binding cooperativity (Esp1396I) was also found to have a lower value of the dissociation constant of dimerization. As a prediction, it will be interesting to test if, in other R-M systems, the value of the dissociation constant of dimerization and the binding cooperativity are also related in this way.

Overall, this work provides an example that the system properties that may appear “random” or even surprising (such as the extremely large binding cooperativity) may be explained by constraints imposed by few general principles (in this case the system dynamical properties). Additionally, some of these system properties may serve other functions, e.g. the leaderless C transcripts might be related with a need for preferential translation under specific physiological conditions [42]. Analyzing other R-M systems can further test relation of the system features with the simple dynamical properties, where the main obstacle is that their transcription regulation is generally not well studied. In particular, investigating up to now poorly understood linear R-M systems, which have different architecture compared to the convergent and the divergent systems studied here, and which do not encode C proteins – but may exhibit control by antisense RNAs or at the level of translation initiation efficiency - may be particularly useful [43, 44]. As a further outlook, it will be interesting investigating if properties of other bacterial immune systems, such as recently discovered CRISPR/Cas systems [45], can also be explained by similar dynamical properties [34]. With that respect note that CRISPR/Cas is more advanced, i.e. adaptive bacterial immune system, which retains a memory of the past infections incorporated as spacers in the CRISPR array [46].

Also, in this work we follow a standard approach in molecular biology, where features of the system are perturbed/mutated (which is here done in-silico), and the effect of these perturbations on the presumed system function is assessed. In addition to such “single mutations”, a computational equivalent of “double” or “triple” mutations can be exhibited, where more than one system feature would be simultaneously perturbed. This would address the question if perturbations in one feature, can be rescued by also perturbing the other feature(s), which is related to the system robustness. While this question is out of the scope of this work, it also provides an interesting outlook for future research.

Finally, the recent advancement of experimental techniques, such as single-cell experiments, allows directly observing the protein dynamics during the system establishment. While in principle arduous, it would be interesting to experimentally observe how the relevant dynamics is perturbed when some of the key system features are abolished. This would then directly put to test some of the prediction from the computational modelling, which we provided here.

## Additional files

Additional file 1: Model of AhdI regulation and dynamics. (PDF 415 kb)

Additional file 2: Model of EcoRV regulation and dynamics. (DOCX 198 kb)

## Abbreviations

C: Control protein
M: Methyltransferase
R: Restriction enzyme
R-M: Restriction-modification
RNAP: RNA polymerase
wt: Wild-type
