# Supplementary material for: Understanding key features of bacterial restriction-modification systems through quantitative modeling
Source: BMC Syst Biol. 2017 Feb 24;11(Suppl 1):1–15. doi: 10.1186/s12918-016-0377-x (PMC5333194; doi:10.1186/s12918-016-0377-x)
Supplement: Supplementary file 1 — Model of AhdI regulation and dynamics. (PDF 415 kb) [file 12918_2016_377_MOESM1_ESM.pdf]

Model of AhdI regulation and dynamics

Thermodynamic model of CR promoter transcription regulation

The model of wild-type AhdI transcription control follows the same assumptions as in [1], for which it was shown that it can well reproduce the experimentally measured dependence of transcription activity on C concentration in wild type system. We start from these assumptions, and from the system parameters determined for the wild type, to systematically (*in-silico*) mutate the main system features, as described below.

Transcription regulation of the CR promoter by C is described by the following set of reactions (shown in Fig. 2):

$$Mon + Mon \underset{K_1}{\rightleftarrows} D \tag{1.1}$$

$$DNA + RNAP \underset{K_2}{\rightleftarrows} RNAP - DNA \tag{1.2}$$

$$D + DNA \underset{K_3}{\rightleftarrows} D - DNA \tag{1.3}$$

$$D - DNA + D \underset{K_4}{\rightleftarrows} T - DNA \tag{1.4}$$

$$D - DNA + RNAP \underset{K_5}{\rightleftarrows} D - DNA - RNAP \tag{1.5}$$

In the above reactions, *Mon*, *D* and *T* denote C monomers, dimers and tetramers, respectively. Reactions given by (1.1)-(1.5) represent:

(1.1) – dimerization of C monomers (Mon);

(1.2) – *RNAP* binding to the CR promoter forming a $RNAP - DNA$ complex;

(1.3) – *D* binding to the distal (high affinity) binding site, forming a $D - DNA$ complex; Note that this configuration was neglected in [1], due to a very low probability of detecting a single dimer bound in the wt AhdI. We here include this configuration, since as we perturb (*in-silico* mutate) the system this configuration can become important.

(1.4) – a second *D* recruited to the proximal (low affinity) binding site, forming a C tetramer on the promoter ($T - DNA$ complex);

(1.5) – *RNAP* recruited to its binding site forming a $D - DNA - RNAP$ complex.

In equilibrium, the above reactions lead to the following relationships between the relevant concentrations and the dissociation constants (note that the square brackets refers to the reactant concentration):

$$K_1 = \frac{[Mon]^2}{[D]} = k^{-1} e^{\Delta G_D} \tag{1.6}$$

$$K_2 = \frac{[DNA] \cdot [RNAP]}{[RNAP - DNA]} = k^{-1} e^{\Delta G_{RNAP(CR)}} \tag{1.7}$$

$$K_3 = \frac{[D] \cdot [DNA]}{[D - DNA]} = k^{-1} e^{\Delta G_L} \tag{1.8}$$

$$K_4 = \frac{[D-DNA]\cdot[D]}{[T-DNA]} = k^{-1}\, e^{\Delta G_R + \Delta G_T} \tag{1.9}$$

$$K_5 = \frac{[D-DNA]\cdot[RNAP]}{[D-DNA-RNAP]} = k^{-1}\, e^{\Delta G_{RNAP(CR)} + \Delta G_{D-RNAP}} \tag{1.10}$$

In the above relations, ΔG_D corresponds to the free energy of C protein dimerization, $\Delta G_{RNAP(CR)}$ to the free energy of RNAP binding to CR promoter, ΔG_L to the free energy of a C dimer binding to the distal binding site, ΔG_R to the free energy of a C dimer binding to the proximal binding site, ΔG_T to the free energy of interaction between the C dimers bound to the distal and the proximal binding site, and ΔG_{D-RNAP} to the free energy of interaction between the C dimer bound to the distal binding site and RNA polymerase (RNAP), with a proportionality constant *k* (in units of concentration [2]).

The model of transcription regulation is based on the assumption by Shea-Ackers [3], by which the promoter transcription activity is proportional to its equilibrium occupancy by RNAP:

$$\varphi_r = \alpha \frac{Z_{RNAP} + Z_{D-RNAP}}{1 + Z_{RNAP} + Z_L + Z_{D-RNAP} + Z_T}. \tag{1.11}$$

Here α is a proportionality constant, while 1, Z_{RNAP}, Z_L, $Z_{D\text{-}RNAP}$ and Z_T denote statistical weights of allowed protein configurations on CR promoter, which are related with the concentrations of appropriate protein-DNA complexes (see reactions and Fig. 2A). That is, *i*) 1 is a statistical weight of an empty promoter, *ii*) $Z_{RNAP} = \left[RNAP-DNA\right]/\left[DNA\right]$ corresponds to the basal transcription, i.e. to only RNAP bound to the promoter, *iii*) $Z_L = \left[D-DNA\right]/\left[DNA\right]$ corresponds to the C dimer bound to its distal binding site, *iv*) $Z_{D-RNAP} = \left[D-DNA-RNAP\right]/\left[DNA\right]$ corresponds to the RNAP recruited to the promoter by the C dimer bound to the distal binding site, and *v*) $Z_T = \left[T-DNA\right]/\left[DNA\right]$ corresponds to C tetramer bound to the promoter.

By using (1.6)-(1.10) with the above statistical weights, Eq. (1.11) becomes:

$$\varphi_r\left(Mon\right) = \alpha \frac{a + b\left[Mon\right]^2}{1 + a + f\left[Mon\right]^2 + b\left[Mon\right]^2 + c\left[Mon\right]^4}. \tag{1.12}$$

Constants a, b, c and *f* above depend on the equilibrium dissociation constants (or corresponding free energies of protein-DNA and protein-protein interactions), and RNAP concentration:

$$a = \frac{\left[RNAP\right]}{K_2} = k\left[RNAP\right]e^{-\Delta G_{RNAP(CR)}} \tag{1.13}$$

$$f = \frac{1}{K_1 K_3} = k^2 e^{-\Delta G_L - \Delta G_D} \tag{1.14}$$

$$b = \frac{[RNAP]}{K_1 K_3 K_5} = k^3 [RNAP] \omega' e^{-\Delta G_L - \Delta G_{RNAP(CR)} - \Delta G_D} \quad (1.15)$$

$$c = \frac{1}{K_1{}^2 K_3 K_4} = k^4 \omega e^{-\Delta G_L - \Delta G_R - 2\Delta G_D}. \quad (1.16)$$

Here, $\omega = \exp(-\Delta G_T)$, corresponds to cooperativity of the dimers binding to the distal and the proximal binding site, while $\omega' = \exp(-\Delta G_{D-RNAP})$ quantifies the strength of RNAP recruiting to the promoter by a C dimer bound to the distal binding site. Note that for wt system, the dissociation constant for C protein dimerization K_1 was shown to be very high, so that almost all C protein in the solution is in the form of monomers, i.e. $[Mon] = [C]$. We will subsequently explore the system dynamics when K_1 is perturbed (decreased), which leads to both monomers and dimers present in the solution (see below).

Thermodynamical model of M promoter transcription regulation

Methyltransferase (M) methylates specific sites in M promoter, which overlap RNAP binding site, thereby repressing transcription of its own gene – this mechanism is also found in other R-M systems (reviewed in [4]). We model the control of M expression as an equilibrium negative autoregulation, as described below.

Transcription regulation of M promoter is then described by the following set of reactions (see Figure 2B):

$$RNAP + DNA \underset{K_{1M}}{\rightleftarrows} RNAP - DNA \quad (1.17)$$

$$M + DNA \underset{K_{2M}}{\rightleftarrows} M - DNA \quad (1.18)$$

The reactions above lead to the following relation between the dissociation constants and the reactant concentrations:

$$K_{1M} = \frac{[RNAP][DNA]}{[RNAP - DNA]} \quad (1.19)$$

$$K_{2M} = \frac{[M][DNA]}{[M - DNA]} \quad (1.20)$$

We again assume that transcription activity is proportional to the promoter occupancy by RNAP:

$$\varphi_m = \beta \frac{Z_{RNAP(M)}}{1 + Z_{RNAP(M)} + Z_M} \quad (1.21)$$

where β is a proportionality constant, and *i*) 1 is the statistical weight for the empty promoter configuration, *ii*) $Z_{RNAP(M)} = [RNAP - DNA]/[DNA]$ is the statistical weight for the configuration with bound RNAP, *iii*) $Z_M = [M - DNA]/[DNA]$ is the statistical weight of M bound to the

promoter. If we use Eqs. (1.19)-(1.20) in the expressions for the statistical weights, and introduce $\varphi_{m(bas)} = \left(\beta \frac{[RNAP]}{K_{1M}} K_{2M} \right) \Big/ K_D$ and $K_D = \left(1 + \frac{RNAP}{K_{1M}} \right) K_{2M}$ (following the notation in [1], we obtain ($\varphi_{m(bas)}$ is the basal transcription rate of M promoter):

$$\varphi_m(M) = \varphi_{m(bas)} \frac{K_D}{K_D + [M]}. \tag{1.22}$$

Next, the numerical values of the parameters in Eqs. (1.12) and (1.22) are determined as follows: α and $\varphi_{m(baz)}$ are adjusted so that, in equilibrium, transcription activities of CR and M promoters become equal (and correspond to 1) [1]. Parameters a, b and c were obtained from the best fit to the experimentally measured transcription activity *vs.* C from the wt system [1], while f was estimated from the dissociation constants measured in [5]:

$$f = \frac{1}{K_1 K_3} = \sqrt{c \frac{K_4}{K_3}} \tag{1.23}$$

Dynamical model of transcript and protein expression

The equations for transcription activity (Eqs. (1.12) and (1.22)) are used as an input in a kinetic model, which describes how R and M transcript and protein concentrations change with time, during establishment of the AhdI R-M system in a naïve host cell. CR operon and M gene transcripts dynamics is modeled by the following differential equations:

$$\frac{dr}{dt} = \varphi_r(Mon) - \lambda_r r \tag{1.24}$$

$$\frac{dm}{dt} = \varphi_m(M) - \lambda_m m \tag{1.25}$$

In both equations, the first term on the right-hand side represents transcript synthesis by transcription of the appropriate genes from their promoters, while the second term represents transcript decay by degradation. Protein dynamics is modeled by a similar set of differential equations shown below, in which the first term on the right-hand side describes protein synthesis by transcript translation, while the second term describes protein decay by degradation (for the notation and the constant values, see the Table 1 below):

$$\frac{dC}{dt} = k_C r - \lambda_C C \tag{1.26}$$

$$\frac{dR}{dt} = k_R r - \lambda_R R \tag{1.27}$$

$$\frac{dM}{dt} = k_M m - \lambda_M M \tag{1.28}$$

C and R are produced by translation of the same CR operon transcript, and as their degradation rates are assumed to be the same, $R = (k_R / k_C)C,$ which reduces the number of the differential

equations to be solved. Note that C transcript is leaderless, so that following [1] $k_R = k_M = 5k_C$ (see Table 1 below).

The system of differential equations (1.24)-(1.26) and (1.28) is numerically solved in MATLAB, using Runge-Kutta method with the initial conditions set to zero. The model of AhdI regulation is next used to assess the changes of the system dynamics, upon gradually abolishing the characteristic features of the system regulation.

**Table 1. Notations used in model equations**		
**Variables**	**Description**	
φ_r	Transcription activity of CR promoter	
φ_m	Transcription activity of M promoter	
r	Concentration of CR operon transcript	
m	Concentration of M gene transcript	
M	Concentration of methyltransferase	
C	Concentration of control protein	
R	Concentration of restriction endonuclease	
**Constants**	**Description**	**Values**
α	Proportionality constant for CR promoter	1.7
$\phi_{baz(m)}$	Basal transcription activity of M promoter	1.7
a	Constants that absorb the relevant interaction energies and RNAP concentration (Eqs. (1.13)-(1.16))	$1.6 \cdot 10^{-1}$
f		$2.8 \cdot 10^{-4}$
b		$1.5 \cdot 10^{-1}$
c		$1.2 \cdot 10^{-5}$
K_D		$6.5 \cdot 10^{2}$
k_C	Translation constant for control protein	$6.0 \cdot 10^{-1}$
k_R	Translation constant for restriction endonuclease	3.0
k_M	Translation constant for methyltransferase	3.0
λ_r	Rate of CR transcript decay	$2.0 \cdot 10^{-1}$
λ_m	Rate of M transcript decay	$2.0 \cdot 10^{-1}$
λ_C	Rate of control protein decay	$3.3 \cdot 10^{-2}$
λ_R	Rate of restriction endonuclease decay	$3.3 \cdot 10^{-2}$
λ_M	Rate of methyltransferase decay	$3.3 \cdot 10^{-2}$

Table 1: Values of the constants are the same as in [1], which were shown to be able to explain the equilibrium measurements for AhdI wild type system. The transcription activities and the transcript decay rates are given in units of transcripts per minute, while the translation initiation rates and the protein decay rates are given in proteins per minute.

Decreasing the dissociation constant of dimerization

We vary the equilibrium dissociation constant of dimerization K_1, which changes the balance of monomers and dimers in the solution. The total concentration of the synthesized C protein is:

$$\left[C\right] = \left[Mon\right] + 2\left[D\right], \tag{1.29}$$

which together with (1.6) leads to:

$$\left[Mon\right] = \frac{K_1}{4}\left(\sqrt{1+\frac{8\left[C\right]}{K_1}} - 1\right) \tag{1.30}$$

In addition to C monomer concentration, f, b and c also depend on K_1 (see Eqs. (1.13)-(1.16)).

One should note two limiting cases when mostly: *i*) monomers are present in the solution $\left[C\right] \approx \left[Mon\right]$, and *ii*) dimers are present in the solution $\left[C\right] \approx 2\left[D\right]$. The first case corresponds to the wt system behavior, with very high K_1, which we estimate as the lowest K_1 in which the monomer dimer balance (Eq. (1.30)) leads to the R dynamics that does not notably depart from the one in wt system (estimated as $K_{1\max} \sim 4000\,\text{nM}$).

The second limiting case occurs for low K_1, when mostly dimers are in the solution, which we derive as follows. We define $K_1' = K_1 / x$ ($x > 1$), so that f, b and c change as:

$$f' = fx, \quad b' = bx, \quad c' = cx^2 \tag{1.31}$$

For the condition $K_1' \ll \left[C\right]$, Eq. (1.30) reduces to $\left[Mon\right] \approx \sqrt{K_1\left[C\right]/2}$, which, together with f', b' and c', transforms Eq. (1.12) to the limit when mostly C dimers are present in the solution:

$$\varphi_r\left(C\right) = \alpha \frac{a + \frac{bK_1}{2}\left[C\right]}{1 + a + \frac{fK_1}{2}\left[C\right] + \frac{bK_1}{2}\left[C\right] + \frac{cK_1^2}{4}\left[C\right]^2} \tag{1.32}$$

Different monomer/dimer ratios in solution, i.e. the cases between these two limits, are obtained by gradually increasing x and solving Eq. (1.30) for $\left[Mon\right]$, which is used together with f', b' and c' (Eq. (1.31)) to calculate the transcription activity (Eq. (1.12)).

Changing cooperativity in the dimer binding

Cooperativity in the dimer binding is decreased by varying ω (see Eq. (1.16)), which translates to varying the parameter c that is related to the establishment of the repressing tetramer complex. As it is estimated that $\omega \sim 3000$ in the wt AhdI system [5], we varied c in the range from $\omega = 3000$ to $\omega = 1$ (the absence of cooperativity).

Changing C protein translation rate

The translation rate for C protein k_C is increased from the initial value, to the same translation rate as for R and M (i.e. k_C is increased two, three, four and five times), while leaving the other parameters unchanged.

The effects on stability of R steady-state levels

In the equilibrium $dr/dt = dC/dt = 0,$ leading to (see Eqs. (1.24) and (1.26)):

$$\varphi_r\left(C_{eq}\right) = \frac{\lambda_r \lambda_C}{k_C} C_{eq} \tag{1.33}$$

Consequently, C_{eq} is found at the intersection of $\varphi_r(C)$ curve given by Eq. (1.12), and the straight line given by the right side of Eq. (1.33). The steady-state stability can be approximated by the value of the spring constant in the oscillator equation derived in the Supplementary files of [1]:

$$\Omega^2 = \lambda_r \lambda_C - k_C \left.\frac{d\varphi_r\left(C\right)}{dC}\right|_{C_{eq}}, \tag{1.34}$$

where positive Ω^2 indicates the stable equilibrium. To quantify the changes in the stability of R steady-state levels, we assess how Ω^2 is changed upon introducing a perturbation in the system (e.g. as K_1 is decreased). Note that the decrease of Ω^2 indicates the decrease in the stability of the R steady-state levels, and vice versa.

The effect on the system switch-like behavior

We here assess how fast the system makes a transition from "OFF" to "ON" state, i.e. from the initial small R values to the values approaching the system equilibrium. For quantifying this effect, we determine the maximum rate of $R(t)$ change during the system evolution. As $R(t)$ curves have sigmoidal shape, this maximal rate corresponds to the time interval in which R makes an approximately linear increase from small to high concentrations.

The effect on the delay of R with respect to M

To quantify the effect of changing the system features on the delay in expression of R with respect to M in the initial interval of the protein synthesis (taken as the first 10 minutes), we define the following parameter (relative delay):

$$\tau_{delay} = \left(\int_1^{10} M(t)dt - \int_1^{10} R_{mut}(t) \right) \Big/ \left(\int_1^{10} M(t)dt - \int_1^{10} R_{wt}(t) \right), \qquad (1.35)$$

where $R_{mut}(t)$ and $R_{wt}(t)$ correspond to the perturbed and the wt system, respectively. Note that, for the wt system, the relative delay is 1, while values larger than 1 indicate an increase in R delay, and vice-verse.

References

1. Bogdanova E, Djordjevic M, Papapanagiotou I, Heyduk T, Kneale G, Severinov K: **Transcription regulation of the type II restriction-modification system AhdI**. *Nucleic Acids Res* 2008, **36**(5):1429-1442.
2. Sneppen K, Giovanni Z: **Physics in Molecular Biology**. Cambridge: Cambridge University Press 2005.
3. Shea MA, Ackers GK: **The OR control system of bacteriophage lambda. A physical-chemical model for gene regulation**. *J Mol Biol* 1985, **181**(2):211-230.
4. Nagornykh M, Bogdanova E, Protsenko A, Solonin A, Zakharova M, Severinov K: **Regulation of gene expression in a type II restriction-modification system**. *Russian Journal of Genetics* 2008, **44**(5):523-532.
5. McGeehan JE, Papapanagiotou I, Streeter SD, Kneale GG: **Cooperative binding of the C.AhdI controller protein to the C/R promoter and its role in endonuclease gene expression**. *J Mol Biol* 2006, **358**(2):523-531.
