# Supplementary material for: Understanding key features of bacterial restriction-modification systems through quantitative modeling
Source: BMC Syst Biol. 2017 Feb 24;11(Suppl 1):1–15. doi: 10.1186/s12918-016-0377-x (PMC5333194; doi:10.1186/s12918-016-0377-x)
Supplement: Supplementary file 2 — Model of EcoRV regulation and dynamics. (DOCX 198 kb) [file 12918_2016_377_MOESM2_ESM.docx]

**Model of EcoRV regulation and dynamics**

**Modeling transcription activity of divergent M and CR promoters**

We here develop the first model of EcoRV transcription regulation and dynamics. We model transcription activity of CR and M promoters, by using the set of reactions schematically shown in Fig. 3:

As in the previous section, *Mon*, *D* and *T* denote C monomers, dimers and tetramers, respectively. Reactions given by - represent:

– dimerization of C monomers;

– *RNAP* binding to the CR promoter forming *RNAP-DNA_R_* complex; note that subscript R denotes the rightward transcription direction (see Figure 3).

– *D* binding to the distal binding site forming *D-DNA* complex;

– *RNAP* recruitment to the CR promoter forming *D-DNA-RNAP_R_* complex;

– *D* recruitment to the proximal binding site forming *T-DNA* complex;

– *RNAP* binding to the *M* promoter forming *RNAP-DNA_L_* complex; subscript L denotes the leftward transcription orientation (see Figure 3).

– *D* binding to the distal binding site forming *RNAP-DNA-D_L_* complex;

– *D* recruitment to the proximal binding site forming *RNAP-DNA-T_L_* complex*.*

In equilibrium, the reactions above lead to the following relations:

$$K_{2R}=\frac{[DNA]\cdot[RNAP]}{[RNAP-DNA]_{R}}$$

$$K_{3}=\frac{[D]\cdot[DNA]}{[D-DNA]}=\frac{[RNAP-DNA]_{L}\cdot[D]}{[RNAP-DNA-D]_{L}}$$

$$\frac{K_{2R}}{\omega'}=\frac{[D-DNA]\cdot[RNAP]}{[D-DNA-RNAP]_{R}}$$

$$\frac{K_{4}}{\omega}=\frac{[D-DNA]\cdot[D]}{[T-DNA]}=\frac{[D-DNA-RNAP]_{L}\cdot[D]}{[RNAP-DNA-T]_{L}}$$

In these equations, $\omega'\equiv e^{-\Delta G_{D-RNAP}^{R}}$corresponds to binding cooperativity between D bound to the distal binding site and *RNAP* bound to CR-promoter, while $\omega\equiv e^{-\Delta G_{D-D}}$ corresponds to the cooperativity of C dimers binding to the distal and the proximal binding site, with $\Delta G_{D-RNAP}$ and $\Delta G_{D-D}$the corresponding energies of the protein-protein interactions.

By again applying the assumption of transcription activity being proportional to occupancy of the promoter by RNA polymerase [[1](#_ENREF_1)], we obtain:

*k* is a proportionality constant, while, similarly as for the AhdI, statistical weights of the allowed protein configurations on the CR and M promoters are denoted by$1, Z_{RNAP_{R}} , Z_{D}, Z_{T}, Z_{D-RNAP_{R}} , Z_{RNAP_{L}} ,$ $Z_{RNAP-D_{L}}$and$Z_{RNAP-T_{L}}.$Here,$Z_{RNAP {}_{R}}=\left[RNAP-DNA\right]_{R}/\left[DNA\right],$ $Z_{D}=\left[D-DNA\right]/\left[DNA\right],$ $Z_{T}=\left[T-DNA\right]/\left[DNA\right],$ $Z_{D-RNAP_{R}}=\left[D-DNA-RNAP\right]_{R}/\left[DNA\right],$ $Z_{RNAP {}_{L}}=\left[RNAP-DNA\right]_{L}/\left[DNA\right],$ $Z_{RNAP-D_{L}}=\left[RNAP-DNA-D\right]_{L}/\left[DNA\right]$ and $Z_{RNAP-T_{L}}=\left[RNAP-DNA-T\right]_{L}/\left[DNA\right].$

We further take into account that C binds to the distal binding site with approximately five times higher affinity compared to the proximal binding site, i.e.$K_{4}/K_{3}=5$[[2](#_ENREF_2)]. If we also take into account that the CR promoter is significantly weaker than the M promoter$K_{2R}\gg K_{2L}$, the transcription activities become:

We above introduced a new constant $x=\frac{\alpha_{L}}{\alpha_{R}}$, where $\alpha_{R}=\frac{[RNAP]}{K_{2R}}, \alpha_{L}=\frac{[RNAP]}{K_{2L}}.$ In accordance with $K_{2R}\gg K_{2L}$, we also used the approximation $\alpha_{L}\gg\alpha_{R}.$

**Modeling the dynamics of CR and M transcripts' and proteins' expressions**

The time dependencies of C, R and M transcript and protein amounts upon EcoRV introduction in a naïve bacterial host is described by the following differential equations:

$$\frac{dC(\mathrm{t})}{dt}=k_{C}r(\mathrm{t})-\lambda_{C}\mathrm{C}(\mathrm{t})$$

$\frac{dM(\mathrm{t})}{dt}=k_{M}\mathrm{m}(\mathrm{t})-\lambda_{M}\mathrm{M}(\mathrm{t})$,

where r and m denote, respectively, CR and M transcript concentrations, while C and M denote, respectively, C and M protein concentrations. Constants $\lambda$(with different subscripts) denote transcript and protein decay rates, while constants *k* (also with different subscripts) denote the translation rates. The first terms on the right-hand side represent transcript/protein synthesis by transcription/translation, while the second terms represent transcript/protein decay by degradation. We assume the same decay rate for the transcripts (1/5 1/min), as well as the same decay rate for all the proteins (1/30 1/min), leading to$R=k_{R} / k_{C}C$. Note that due to this relation, we left out the equation for R protein dynamics, i.e. R dynamics is described by Eq. . The above system of differential equations is again solved numerically in MATLAB by Runge-Kutta method, with the initial conditions set to zero.

**Dynamics of wild-type EcoRV**

We apply the model developed above to the wt EcoRV system. We assume that in the solution, most of C proteins are dimers, so we express the transcription activities in terms of D by using Eq. . Since there is no cooperativity in C binding to the distal and the proximal binding site in wt EcoRV (see [[2](#_ENREF_2)]), we also set ω=1 which leads to:

Taking into account that monomer concentration is close to zero, leads to:

$$C=Mon+2D\Rightarrow D\approx C/2$$

Furthermore, to reduce the free parameters, and since the absolute protein amounts are not relevant for the analysis, we introduce the following scaling:

$\overline{C}=\frac{C}{2K_{3}}$, $\overline{k}=\frac{k}{2K_{3}}$, $\overline{K}_{1}=\frac{K_{1}}{16K_{3}}$

Finally, with the rescaling, the transcription activities acquire the form:

where we assume: *x*=10, i.e. an order of magnitude higher M compared to CR basal promoter rate [[2](#_ENREF_2)]; $\omega'=20$, i.e. an order of magnitude smaller strength of protein-protein interactions than found in [[3](#_ENREF_3)], consistently with the absence of cooperativity in EcoRV C dimer binding [[2](#_ENREF_2)]. Furthermore, it was found in [[2](#_ENREF_2)] that the steady-state levels of CR and M transcripts are comparable in the presence of C, which we use to set the value of $\overline{k},$as described below.

Rewriting Eqs. (2.1)-(2.4) in terms of rescaled variables and assuming the same transcript decay rates, the condition of the comparable CR and M transcript amounts leads to the equality of CR and M transcription activities in the equilibrium (when the left-hand sides of Eqs. - are zeros). Eqs. - then lead to:

$$1+\omega'\overline{C}_{eq}=x(1+\overline{\mathrm{C}}_{eq}+\frac{\overline{C}_{eq}^{2}}{5})$$

$\overline{\mathrm{C}}_{eq}$can be determined from the equation above, and consequently,$\overline{k}$can also be obtained by eliminating $\overline{r}$ from rescaled analogs of Eqs. and in the equilibrium, i.e.:

Regarding the translation constants, in the wild-type system we use $k_{M} = k_{R} = k_{C} = 3,$ since *C* transcript is not leaderless [[2](#_ENREF_2)], contrary to AhdI system [[4](#_ENREF_4)]. We further analyze the divergent *R-M* systems, by introducing AhdI characteristic features to wt EcoRV.

**Increasing the dissociation constant of dimerization**

We next gradually increase the (rescaled) dissociation constant of dimerization $\overline{K}_{1}$, from the limiting case in which mostly C dimers are in the solution (corresponding to wt EcoRV), to the other limit in which mostly monomers are in the solution (corresponding to wt AhdI).

We first investigate the gradual transition from the solution containing only C dimers to the solution containing only C monomers, by varying rescaled equilibrium dissociation constant of dimerization$\overline{K}_{1}$. The dependence of C monomers on $\overline{K}_{1}$ is obtained by using the left side of Eq. and the rescaled equivalent of Eq. :

$\frac{Mon^{2}}{K_{1}K_{3}}=\overline{K}_{1}[\sqrt{1+\frac{\overline{C}}{\overline{K}_{1}}}-1]^{2}$ ,

which is then substituted in Eqs. and (with $\omega=1$), where we also introduce $\overline{k}=\frac{k}{2K_{3}}$.

Wild-type transcription activities (Eqs. -) represent the limiting case of mostly dimers $(\overline{K}_{1}\ll\overline{C}).$ To determine$\overline{K}_{1}$ that corresponds to this limit, we search for maximal value of $\overline{K}_{1}$when wt M and R curves nearly overlap with the corresponding curves that result from imposing the monomer dimer balance (Eq. ) (leading to $\overline{K}_{1}$~1/1000).

The other limit of $\overline{K}_{1}\gg\overline{C}$ corresponds to mostly monomers in solution, where Eq. can be Tylor expanded to $Mon^{2} / K_{1} K_{3} \approx\bar{C}^{2} / 4\bar{K}_{1},$ so that rescaled equivalents of Eqs. - lead to:

To estimate $\overline{K}_{1}$ corresponding to this limit, we gradually increase $\overline{K}_{1}$ until M and R curves (that result from the limiting case, i.e. Eqs. -) overlap with the corresponding curves that result from imposing the full monomer dimer balance (Eq. ) (resulting in $\overline{K}_{1}$~10)). To assess how increasing the dissociation constant of dimerization towards the high values characteristic for AhdI changes R and M dynamics, we vary the dissociation constant of dimerization $\overline{K}_{1}$ from the small values corresponding to mostly dimers in the solution to the high values corresponding to the monomer limit.

**Increasing the cooperativity in dimer binding and decreasing the C protein translation rate**

We also assess the changes in R and M dynamics when the high cooperativity in C dimer binding to the promoter is introduced to EcoRV. To that end, we use the rescaled form of Eqs. and , in which we change ω from ω=1 that is found in wt EcoRV, to higher binding cooperativity, by gradually increasing ω (i.e. ω=1, 2, 4, 8, 16). Note that the other wt EcoRV features are unchanged.

Finally, we investigate the effect of introducing lower C translation rate, where we decrease its value from the one equal to that of R and M transcripts (used in EcoRV), to the five times lower value used in AhdI (i.e. *k_C_*=3, 12/5, 9/5, 6/5, 3/5), where we do not change other EcoRV features.

**References**

1. Shea MA, Ackers GK: **The OR control system of bacteriophage lambda. A physical-chemical model for gene regulation**. *J Mol Biol* 1985, **181**(2):211-230.

2. Semenova E, Minakhin L, Bogdanova E, Nagornykh M, Vasilov A, Heyduk T, Solonin A, Zakharova M, Severinov K: **Transcription regulation of the EcoRV restriction-modification system**. *Nucleic Acids Res* 2005, **33**(21):6942-6951.

3. Bogdanova E, Zakharova M, Streeter S, Taylor J, Heyduk T, Kneale G, Severinov K: **Transcription regulation of restriction-modification system Esp1396I**. *Nucleic acids research* 2009, **37**(10):3354-3366.

4. Bogdanova E, Djordjevic M, Papapanagiotou I, Heyduk T, Kneale G, Severinov K: **Transcription regulation of the type II restriction-modification system AhdI**. *Nucleic Acids Res* 2008, **36**(5):1429-1442.
